# Health care utilisation for treatment of injuries among immigrants in Norway: a nationwide register linkage study

**DOI:** 10.1186/s40621-020-00286-7

**Published:** 2020-11-16

**Authors:** Eyvind Ohm, Kristin Holvik, Marte Karoline Råberg Kjøllesdal, Christian Madsen

**Affiliations:** Norwegian Institute of Public Health, PO Box 222, Skøyen, 0213 Oslo, Norway

**Keywords:** Injuries, Minority health, Immigrants, Incidence, Secondary care, Primary health care, Data linkage, Registries, Register-based research, International classification of diseases

## Abstract

**Background:**

Previous research has generally found lower rates of injury incidence in immigrant populations than in native-born populations. Most of this literature relies on mortality statistics or hospital data, and we know less about injuries treated in primary health care. The aim of the present study was to assess use of primary and secondary care for treatment of injuries among immigrants in Norway according to geographic origin and type of injury.

**Methods:**

We conducted a nationwide register-based cohort study of all individuals aged 25–64 years who resided in Norway as of January 1st 2008. This cohort was followed through 2014 by linking sociodemographic information and injury data from primary and secondary care. We grouped immigrants into six world regions of origin and identified immigrants from the ten most frequently represented countries of origin. Six categories of injury were defined: fractures, superficial injuries, open wounds, dislocations/sprains/strains, burns and poisoning. Poisson regression models were fitted to estimate incidence rate ratios separately for injuries treated in primary and secondary care according to immigrant status, geographic origin and type of injury, with adjustment for sex, age, county of residence, marital status and socioeconomic status.

**Results:**

Immigrants had a 16% lower incidence of injury in primary care than non-immigrants (adjusted IRR = 0.84, 95% CI 0.83–0.84), and a 10% lower incidence of injury in secondary care (adjusted IRR = 0.90, 95% CI 0.90–0.91). Immigrants from Asia, Africa and European countries outside EU/EEA had lower rates than non-immigrants for injuries treated in both primary and secondary care. Rates were lower in immigrants for most injury types, and in particular for fractures and poisoning. For a subset of injuries treated in secondary care, we found that immigrants had lower rates than non-immigrants for treatment of self-harm, falls, sports injuries and home injuries, but higher rates for treatment of assault, traffic injuries and occupational injuries.

**Conclusions:**

Health care utilisation for treatment of injuries in primary and secondary care in Norway was lower for immigrants compared to non-immigrants. Incidence rates were especially low for immigrants originating from Asia, Africa and European countries outside EU/EEA, and for treatment of fractures, poisoning, self-harm and sports injuries.

## Background

Like many other European countries, Norway has witnessed a rapid growth in immigration in recent decades. Between 1990 and 2020, the proportion of immigrants in Norway increased from 4 to 15% of the total population, with their descendants making up a further 4% (Statistics Norway 2020). In the capital Oslo, one in three inhabitants had an immigrant background as of January 2020. This ongoing demographic change warrants an increased focus on health outcomes of immigrants and their use of health care services (Orcutt et al. 2020).

In this study, we assess one aspect of migrant health in Norway by examining risk of injury. Injuries remain a major public health challenge, being a leading cause of death for young people worldwide and placing a substantial burden on health care services (Polinder et al. 2012; Global Burden of Disease Study 2013 Collaborators 2015; Haagsma et al. 2016; World Health Organisation 2014; Polinder et al. 2016). Research in developed countries comparing injury risk between immigrants and their native-born counterparts has generally found lower incidence rates in immigrant populations (Schwebel et al. 2005; Xiang et al. 2007; Laursen and Moller 2009; Sandvik et al. 2012; Norredam et al. 2013; Karimi et al. 2015; Saunders et al. 2017; Chang and Miller 2018; Andersen and Lauritsen 2020; Aamodt et al. 2020). This advantage occurs despite the presence of various risk factors that ordinarily correlate with worse health outcomes, like lower socioeconomic position (Mackenbach et al. 2008). However, this general finding disguises considerable variation, as injury incidence in immigrant groups differs substantially according to factors such as region/country of origin, reason for migration, length of stay in the host country and type of injury (Laursen and Moller 2009; Sandvik et al. 2012; Norredam et al. 2013; Karimi et al. 2015; Saunders et al. 2017; Aamodt et al. 2020; Saunders et al. 2018).

Most of the literature exploring injury risk in immigrant populations relies on mortality statistics or hospital data. There is less available data about immigrants’ use of primary health care for treatment of injuries. Previous research has shown that a substantial proportion of injured patients in Norway are treated in primary care, either by general practitioners (GPs) or in out-of-hours emergency primary health care (EPHC) (Ohm et al. 2020). This study further showed a different epidemiological pattern for injuries treated exclusively in primary care, in terms of demographic profile (age and gender distributions of injured patients) and the types of injuries that predominate. These findings, combined with indications that some immigrant groups may have limited knowledge about the health care system in their host country and may experience barriers to seeking primary health care (Norredam et al. 2004; Norredam et al. 2007; Straiton and Myhre 2017), suggest there may be differences in the way immigrants use primary care for treatment of injuries, as compared to secondary care and relative to the host population. Evidence of such differences in utilisation may in turn call for strategies that ensure equity in access to and quality of health services.

The main purpose of the current study was to examine injury risk among adult immigrants in Norway, and investigate whether there are differences in their use of primary and secondary care for treatment of injuries. We first included all immigrants in one group and compared incidence rates of injuries treated in primary and secondary care in the period 2008–2014 to those of non-immigrants, taking into account several sociodemographic variables. We further aimed to investigate whether immigrants’ health care utilisation for treatment of injuries varied according to geographic origin and type of injury.

## Methods

### Study design

In this cohort study, we linked sociodemographic information supplied by Statistics Norway with injury diagnoses retrieved from health registers at the individual level using unique personal identification numbers given to all residents.

### Study population

The study population included all individuals who, according to the National Registry, were 25–64 years of age and resided in Norway as of January 1st 2008. We limited the study sample to this age range as younger age groups may not have obtained a stable socioeconomic status, and many older residents receive no labour income. Immigrants in this study were defined as individuals who were born abroad by two foreign-born parents, and were compared to all other residents, here termed non-immigrants. Adopting categories used by Statistics Norway (Statistics Norway 2020), we grouped immigrants into six world regions on the basis of country of origin: 1) European Union (EU) or European Economic Area (EEA) countries, 2) European countries outside EU/EEA, 3) Africa, 4) Asia including Turkey, 5) North America and Oceania and 6) South- and Central America. We subsequently identified immigrants from the ten most frequently represented countries of origin: Denmark, Sweden, Poland, Germany, Bosnia-Herzegovina, Somalia, Iraq, Iran, Pakistan and Vietnam. In sum, immigrants from these ten countries made up 47% of the immigrant population in our cohort.

### Identification of injuries

For injuries treated in primary care, we obtained data from the Norwegian Health Economics Administration database, which contains all electronic reimbursement claims sent by primary care providers in Norway. In this study, only reimbursements from medical doctors were included. Diagnoses in this database are coded according to the second edition of the *International Classification of Primary Care* (ICPC-2). For injuries treated in secondary care, we used data from the Norwegian Patient Registry (NPR), which covers all inpatient, day patient and outpatient specialist health services in Norway. Diagnoses in NPR are coded in accordance with the tenth edition of the *International Statistical Classification of Diseases and Related Health Problems* (ICD-10). Table 1 shows the ICPC-2 and ICD-10 codes used to identify injuries, categorised by the following injury types: fractures, superficial injuries, open wounds, dislocations/sprains/strains, burns and poisoning. Analyses in both primary and secondary care were restricted to contacts where an injury was the principal diagnosis (i.e., the first listed diagnosis).

**Table 1:** ICPC-2 codes (primary care) and ICD-10 codes (secondary care) by injury type

Injury type	ICPC-2	ICD-10
Fractures	L72-L76	S02, S12, S22, S32, S42, S52, S62, S72, S82, S92, T02, T08, T10, T12, T14.2
Superficial injuries	F75, H78, S12, S16, S17, S19	S00, S10, S20, S30, S40, S50, S60, S70, S80, S90, T00, T09.0, T11.0, T13.0, T14.0
Open wounds	S13, S15, S18	S01, S11, S21, S31, S41, S51, S61, S71, S81, S91, T01, T09.1, T11.1, T13.1, T14.1
Dislocations/sprains/strains	L77-L81, L96	S03, S13, S23, S33, S43, S53, S63, S73, S83, S93, T03, T09.2, T11.2, T13.2, T14.3
Burns	S14	T20-T32
Poisoning	A84, A86	T36-T65
Other miscellaneous injuries	A80, A81, A88, B76, B77, D79, D80, F76, F79, H76, H77, H79, N79-N81, R87, R88, U80, X82, Y80	Remaining codes S00-T78

In the period 2009–2014, a subset of the injuries treated in secondary care contains additional information about the external circumstances of the injury (intent, place of occurrence, activity, mechanism etc.). Based on this information, these injuries were further classified into the following categories: self-harm, assault, falls, traffic injuries, occupational injuries, sports injuries and home injuries.

### Covariates

We included the following sociodemographic variables as covariates (all based on information as of January 1st 2008): sex, age, county of residence, marital status and socioeconomic status (SES). County of residence was included to account for potential geographical variation in demographic composition, risk factors and health care utilisation of immigrants, combined with differences in the way emergency care is organised between treatment levels locally. For marital status, we created four categories: married (including registered partner of same-sex marriages), divorced/separated (including divorced/separated partner of same-sex marriages), widow/widower (including surviving partner of same-sex marriages) and unmarried.

Since we had no information on educational attainment for 19% of immigrants in our cohort, we defined socioeconomic status as a composite score of educational attainment and income level (Lampart et al. 2013). We first grouped educational attainment (obtained from the National Education Database) in nine categories according to codes in the Norwegian Standard Classification of Education (Statistics Norway 2016), ranging from no education/preschool education to postgraduate education. We next divided income earned in 2007 into nine quantiles, and then summed these two scores for each individual, yielding a composite score ranging from two to 18. For individuals with missing information on education (2% of the entire cohort), we computed a composite score by multiplying their income score by two. We finally divided this composite score into quintiles.

### Statistical analyses

We first performed descriptive analyses for the sociodemographic variables by immigrant status (immigrants vs. non-immigrants) and region of origin. To obtain person-time at risk, each individual was followed from January 1st 2008 until first registration of an injury diagnosis, date of emigration, date of death or December 31st 2014 (end of follow-up), whichever occurred first. Crude incidence rates were calculated as the number of injury events divided by the sum of person-time at risk. Observation times and incidence rates were calculated separately for injuries treated in primary and secondary care, regardless of injury diagnoses registered in the other treatment level in the same individual. We used Poisson regression models to estimate incidence rate ratios (IRRs) and corresponding 95% confidence intervals (CI) for injury according to immigrant status, adjusting for sex, age, county of residence, marital status and SES. We then estimated IRRs for injury according to region of origin and for ten specific countries of origin, compared with non-immigrants. Subsequently, we analysed differences in health care utilisation according to type of injury, computing IRRs in immigrant groups compared to non-immigrants separately for fractures, superficial injuries, open wounds, dislocations/sprains/strains, burns and poisoning. Finally, for a subset of injuries treated in secondary care where information about external circumstances was available, we computed corresponding IRRs separately for self-harm, assault, falls, traffic injuries, occupational injuries, sports injuries and home injuries.

## Results

The cohort comprised 2,534,434 adults, of whom 10.8% were immigrants (Table 2). Immigrants originating from EU/EEA countries constituted the largest immigrant group (39.3%), followed by immigrants from Asia (34.2%). Compared to non-immigrants, immigrants from EU/EEA countries and Africa were more likely male, whereas immigrants from European countries outside EU/EEA, Asia and South- and Central America had a higher proportion of women. The immigrant population was generally younger, in particular for those originating from Africa and Asia, and more likely to be married compared with non-immigrants. Immigrants from Africa and Asia had the highest proportions of individuals in the lowest quintile of SES score. Immigrants from EU/EEA countries, North America and Oceania had the most favourable socioeconomic profile, and were comparable to non-immigrants on this characteristic. Compared to non-immigrants, immigrants from all regions were more likely to reside in the capital of Oslo, especially for those originating from Africa and Asia.

**Table 2:** Crude sociodemographic characteristics by immigrant status and region of origin. N and distribution (percent)

	Non-immigrants	Immigrants						
		All	EU/EEA countries	European countries outside EU/EEA	Africa	Asia (incl. Turkey)	North America and Oceania	South- and Central America
**N**	2,260,693	273,741	107,603	26,578	28,371	93,732	6480	10,977
**Sex**								
Male	50.8	51.6	57.8	42.4	56.2	46.8	50.5	42.4
**Age**								
25–34	23.0	34.6	32.8	34.8	40.4	35.4	25.1	35.4
35–44	27.6	32.1	29.7	31.6	36.5	34.2	30.0	29.4
45–54	25.5	21.5	21.6	23.6	17.3	21.3	26.3	24.1
55–64	23.8	11.8	15.9	10.0	5.9	9.1	18.6	11.0
**Marital status**								
Married	50.8	63.1	55.0	70.8	56.6	72.4	67.5	56.9
Divorced/separated	13.7	13.7	12.1	12.4	21.0	12.8	13.3	21.9
Widowed	1.5	1.5	1.1	1.8	2.2	1.7	0.8	1.1
Unmarried	34.0	21.8	31.9	14.9	20.2	13.1	18.4	20.2
**SES quintile**								
Q1 (lowest)	23.6	40.3	28.4	36.3	58.0	50.9	24.5	38.4
Q2	18.4	19.6	19.2	23.1	17.5	19.3	18.2	22.7
Q3	18.5	15.5	17.8	18.6	11.3	13.2	15.5	16.9
Q4	26.1	14.9	19.6	15.4	9.0	10.9	19.1	15.1
Q5 (highest)	13.4	9.7	15.0	6.6	4.2	5.7	22.7	6.8
**County of residence**								
Oslo	11.2	29.4	21.5	20.5	45.0	36.7	24.0	29.6

Of the entire cohort, 939,218 individuals (37.1%) were treated for injury in primary care during the follow-up period, while 575,124 (22.7%) were treated for injury in secondary care. For both non-immigrants and immigrants, crude incidence rates were higher for men than for women, but showed no clear pattern by age (Table 3). Married individuals had the lowest and divorced/separated individuals had the highest injury rates. As for SES, rates were lowest for those in the highest quintile, but the socioeconomic gradient was much weaker for immigrants.

**Table 3:** Crude injury incidence^a^ (95% CI) by treatment level, immigrant status and sociodemographic characteristics

	Primary care		Secondary care	
	Non-immigrants	Immigrants	Non-immigrants	Immigrants
**Sex**				
Male	7.8 (7.7–7.8)	6.3 (6.2–6.3)	4.1 (4.1–4.1)	4.3 (4.3–4.4)
Female	6.4 (6.4–6.4)	5.0 (4.9–5.0)	3.5 (3.5–3.5)	3.2 (3.1–3.2)
**Age**				
25–34	7.1 (7.1–7.1)	5.5 (5.4–5.5)	4.0 (3.9–4.0)	3.9 (3.8–3.9)
35–44	7.1 (7.1–7.1)	5.7 (5.6–5.7)	3.7 (3.7–3.7)	3.7 (3.7–3.8)
45–54	7.1 (7.0–7.1)	5.7 (5.7–5.8)	3.7 (3.7–3.7)	3.7 (3.6–3.7)
55–64	7.0 (7.0–7.0)	5.5 (5.4–5.6)	3.9 (3.8–3.9)	3.6 (3.6–3.7)
**Marital status**				
Married	6.9 (6.9–6.9)	5.5 (5.5–5.6)	3.5 (3.5–3.5)	3.5 (3.5–3.6)
Divorced/ separated	7.9 (7.8–7.9)	5.9 (5.8–6.0)	4.4 (4.4–4.4)	4.3 (4.2–4.4)
Widowed	7.3 (7.2–7.4)	5.6 (5.3–5.9)	4.1 (4.0–4.2)	3.7 (3.5–4.0)
Unmarried	7.0 (7.0–7.1)	5.6 (5.6–5.7)	4.0 (4.0–4.1)	4.2 (4.1–4.2)
**SES quintile**				
Q1 (lowest)	7.6 (7.6–7.6)	5.0 (5.0–5.1)	4.3 (4.2–4.3)	3.6 (3.5–3.6)
Q2	7.4 (7.4–7.5)	5.9 (5.8–5.9)	3.8 (3.8–3.8)	3.8 (3.7–3.9)
Q3	7.5 (7.5–7.6)	6.4 (6.3–6.5)	3.8 (3.8–3.9)	4.0 (3.9–4.1)
Q4	7.0 (6.9–7.0)	6.4 (6.3–6.5)	3.6 (3.6–3.7)	4.2 (4.1–4.2)
Q5 (highest)	5.4 (5.4–5.5)	4.9 (4.7–5.0)	3.3 (3.3–3.3)	3.4 (3.3–3.5)

^a^Rates per 100 person-years

**Fig. 1:**
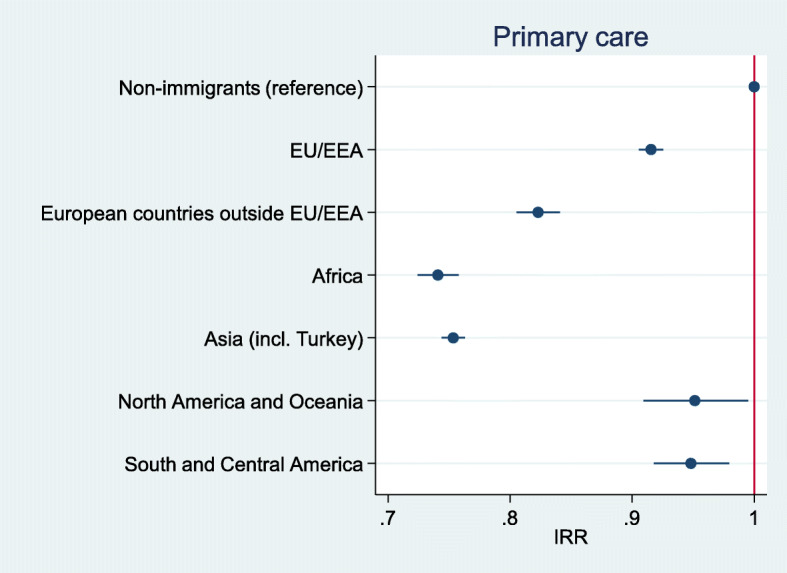
Incidence rate ratios* for injuries treated in primary care, by region of origin. * Adjusted for sex, age, county of residence, marital status and SES

**Fig. 2:**
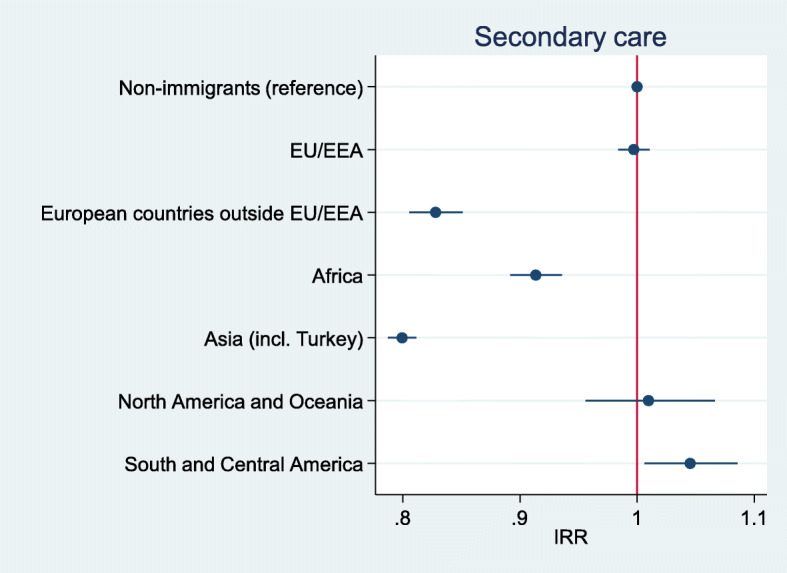
Incidence rate ratios* for injuries treated in secondary care, by region of origin. * Adjusted for sex, age, county of residence, marital status and SES

After adjusting for age, sex, county of residence, marital status and SES, immigrants as a whole had a 16% lower incidence of injury in primary care than non-immigrants (IRR = 0.84, 95% CI 0.83–0.84, *p* < .001), and a 10% lower incidence of injury in secondary care (IRR = 0.90, 95% CI 0.90–0.91, p < .001). Immigrants from European countries outside EU/EEA, Africa and Asia had lower rates than non-immigrants for injuries treated in both primary (Fig. 1) and secondary care (Fig. 2). Immigrants from EU/EEA countries had lower rates than non-immigrants for injuries treated in primary care, but not for injuries treated in secondary care. Incidence rates for immigrants from the Americas and Oceania were comparable to non-immigrants.

Table 4 shows adjusted IRRs for injuries treated in primary and secondary care according to country of origin for ten specific countries, with non-immigrants as the reference. In both primary and secondary care, incidence rates were lowest for immigrants from Vietnam. Immigrants from Bosnia-Herzegovina, Iraq and Pakistan also had low rates. Immigrants from Denmark, Sweden and Germany had higher rates than non-immigrants for injuries treated in secondary care, but not for injuries treated in primary care. For most countries, IRRs were lower for injuries treated in primary care than for injuries treated in secondary care, and this difference was considerable for immigrants from Poland and Somalia.

**Table 4:** Injured patients and incidence rate ratios^a^, by treatment level and country of origin

	N	Injured patients (percent)		IRR (95% CI)	
		Primary care	Secondary care	Primary care	Secondary care
Non-immigrants (reference)	2,260,693	858,313 (38.0)	517,179 (22.9)	1.00	1.00
Denmark	11,642	4041 (34.7)	2702 (23.2)	1.03 (1.00–1.06)	1.12 (1.08–1.16)
Sweden	19,544	5998 (30.7)	4342 (22.2)	0.98 (0.95–1.00)	1.04 (1.01–1.07)
Poland	24,319	7117 (29.3)	5159 (21.2)	0.82 (0.80–0.84)	0.98 (0.96–1.01)
Germany	11,730	3938 (33.6)	2565 (21.9)	0.98 (0.95–1.01)	1.05 (1.01–1.09)
Bosnia-Herzegovina	8765	2918 (33.3)	1638 (18.7)	0.86 (0.83–0.89)	0.80 (0.76–0.84)
Somalia	9170	2561 (27.9)	2469 (26.9)	0.74 (0.72–0.77)	0.97 (0.94–1.01)
Iraq	10,799	3526 (32.7)	2469 (22.9)	0.85 (0.82–0.87)	0.90 (0.87–0.94)
Iran	9215	2979 (32.3)	2330 (25.3)	0.89 (0.86–0.93)	1.01 (0.97–1.05)
Pakistan	12,665	3195 (25.2)	3205 (25.3)	0.78 (0.75–0.80)	0.89 (0.86–0.92)
Vietnam	10,261	2488 (24.2)	1589 (15.5)	0.60 (0.58–0.63)	0.59 (0.57–0.62)

^a^Adjusted for sex, age, county of residence, marital status and SES

### Injury types

Table 5 shows that immigrants had lower incidence rates than non-immigrants for treatment of most injury types, and in particular for poisoning and fractures. Rates for treatment of poisoning were especially low for immigrants originating from Africa, while immigrants from Asia had the lowest rates for treatment of fractures. Burns was the only injury category where immigrants had higher rates than non-immigrants, although only for treatment in secondary care. Rates for treatment of burns were especially high for immigrants from Asia. For most injury types, IRRs in immigrant groups combined compared with non-immigrants were lower for injuries treated in primary care than for injuries treated in secondary care. This difference was most pronounced for treatment of superficial injuries and open wounds (Table 5). For fractures and dislocations/sprains/strains, there was little difference in IRRs between treatment levels.

**Table 5:** Incidence rate ratios^a^ (95% CI) by injury type, treatment level and immigrant background

	Fractures		Superficial injuries		Open wounds		Dislocations/sprains/strains		Burns		Poisoning	
	Primary care	Secondary care	Primary care	Secondary care	Primary care	Secondary care	Primary care	Secondary care	Primary care	Secondary care	Primary care	Secondary care
Injured patients	216,258	234,823	148,755	135,293	284,441	81,495	370,425	124,978	26,354	9260	17,150	15,663
**Immigrant status**												
Non-immigrants (reference)	1.00	1.00	1.00	1.00	1.00	1.00	1.00	1.00	1.00	1.00	1.00	1.00
All immigrants	0.81 (0.79–0.82)	0.80 (0.79–0.81)	0.77 (0.76–0.79)	1.00 (0.98–1.02)	0.67 (0.66–0.68)	0.92 (0.90–0.94)	0.94 (0.93–0.95)	0.95 (0.93–0.97)	1.01 (0.97–1.05)	1.24 (1.16–1.32)	0.65 (0.62–0.69)	0.56 (0.53–0.59)
**Region**												
EU/EEA	0.97 (0.94–0.99)	0.98 (0.96–1.00)	0.84 (0.81–0.86)	1.00 (0.97–1.03)	0.82 (0.80–0.84)	1.03 (0.99–1.06)	0.99 (0.97–1.01)	1.04 (1.01–1.07)	0.90 (0.84–0.96)	1.09 (0.99–1.21)	0.66 (0.61–0.73)	0.60 (0.55–0.66)
European countries outside EU/EEA	0.79 (0.76–0.83)	0.75 (0.72–0.79)	0.75 (0.71–0.80)	0.83 (0.79–0.88)	0.65 (0.63–0.68)	0.83 (0.77–0.89)	0.91 (0.88–0.94)	0.86 (0.81–0.91)	1.11 (1.00–1.24)	1.24 (1.04–1.49)	0.67 (0.57–0.79)	0.52 (0.43–0.62)
Africa	0.74 (0.70–0.78)	0.76 (0.72–0.79)	0.60 (0.56–0.64)	1.15 (1.10–1.20)	0.48 (0.46–0.51)	0.85 (0.80–0.89)	0.89 (0.86–0.92)	0.98 (0.94–1.03)	1.01 (0.90–1.13)	1.28 (1.11–1.49)	0.55 (0.48–0.64)	0.37 (0.32–0.44)
Asia (incl. Turkey)	0.63 (0.62–0.65)	0.61 (0.59–0.62)	0.71 (0.69–0.74)	0.97 (0.94–0.99)	0.53 (0.52–0.55)	0.83 (0.80–0.86)	0.87 (0.85–0.89)	0.82 (0.80–0.85)	1.10 (1.03–1.17)	1.39 (1.27–1.52)	0.66 (0.60–0.71)	0.59 (0.54–0.64)
North America and Oceania	0.98 (0.90–1.08)	0.95 (0.87–1.04)	0.89 (0.79–1.00)	1.14 (1.02–1.27)	0.90 (0.83–0.98)	1.06 (0.92–1.21)	1.04 (0.97–1.12)	1.13 (1.01–1.26)	1.03 (0.78–1.35)	0.71 (0.42–1.20)	0.51 (0.32–0.82)	0.59 (0.37–0.92)
South- and Central America	0.79 (0.73–0.85)	0.81 (0.76–0.87)	0.97 (0.89–1.05)	1.17 (1.09–1.26)	0.66 (0.62–0.71)	1.17 (1.07–1.27)	1.17 (1.11–1.23)	1.27 (1.18–1.36)	0.93 (0.76–1.12)	1.33 (1.04–1.71)	0.89 (0.73–1.10)	0.71 7(0.57–0.90)

^a^Adjusted for sex, age, county of residence, marital status and SES

Information about external circumstances was available for 23.3% (*N* = 133,928) of all patients treated for injury in secondary care. Table 6 shows adjusted IRRs for this subset by injury type. Incidence rates for immigrants were lowest for treatment of injuries caused by self-harm, with a 57% lower risk compared to non-immigrants (IRR = 0.43, 95% CI 0.36–0.53, *p* < .001). Immigrants also had lower incidence rates than non-immigrants for treatment of falls, sports injuries and home injuries. Higher rates for immigrants than non-immigrants were observed for treatment of injuries caused by assault, traffic injuries and occupational injuries. Rates for treatment of injuries caused by assault were especially high for immigrants originating from Africa, with a twofold higher risk compared to non-immigrants.

**Table 6:** Incidence rate ratios^a^ (95% CI) by injury type^b^ and immigrant background

	Intentional injuries		Unintentional injuries				
	Self-harm	Assault	Falls	Traffic injuries	Occupational injuries	Sports injuries	Home injuries
N	1314	6625	65,390	30,237	12,464	13,551	46,008
**Immigrant status**							
Non-immigrants (reference)	1.00	1.00	1.00	1.00	1.00	1.00	1.00
All immigrants	0.43 (0.36–0.53)	1.41 (1.33–1.50)	0.94 (0.92–0.96)	1.15 (1.11–1.18)	1.67 (1.59–1.75)	0.73 (0.69–0.77)	0.96 (0.93–0.98)
**Region**							
EU/EEA	0.44 (0.31–0.62)	0.86 (0.76–0.96)	1.00 (0.96–1.04)	0.97 (0.92–1.02)	1.79 (1.68–1.91)	0.84 (0.77–0.91)	0.98 (0.94–1.02)
European countries outside EU/EEA	0.41 (0.21–0.79)	1.26 (1.05–1.52)	0.79 (0.73–0.85)	1.01 (0.91–1.11)	1.49 (1.30–1.70)	0.63 (0.52–0.76)	0.86 (0.79–0.93)
Africa	0.29 (0.18–0.49)	2.29 (2.09–2.50)	1.15 (1.09–1.21)	1.66 (1.57–1.75)	1.74 (1.57–1.94)	0.67 (0.57–0.78)	0.99 (0.94–1.05)
Asia (incl. Turkey)	0.48 (0.36–0.63)	1.49 (1.37–1.62)	0.83 (0.80–0.86)	1.13 (1.08–1.18)	1.56 (1.45–1.67)	0.59 (0.53–0.65)	0.93 (0.89–0.96)
North America and Oceania	0.83 (0.27–2.59)	0.39 (0.18–0.81)	1.00 (0.87–1.15)	1.04 (0.85–1.26)	1.34 (0.98–1.83)	0.84 (0.61–1.17)	1.10 (0.94–1.29)
South- and Central America	0.48 (0.21–1.07)	1.83 (1.51–2.23)	1.12 (1.02–1.22)	1.29 (1.15–1.44)	1.71 (1.42–2.06)	1.35 (1.12–1.63)	1.06 (0.96–1.17)

^a^Adjusted for sex, age, county of residence, marital status and SES

^b^In a subset of injuries (*N* = 133,928) treated in secondary care in the period 2009–2014 with available information on external circumstances

## Discussion

To our knowledge, this register study is the first to include the full spectrum of medically treated injuries to explore injury risk in immigrant populations. The results show both similarities and differences in the way adult immigrants in Norway seek primary and secondary care for treatment of injuries. Overall, observed injury incidence was lower for immigrants than non-immigrants both for injuries treated in primary and secondary care. Furthermore, the risk of injury by geographic origin also revealed some degree of consistency across treatment level, with immigrants originating from Asia, Africa and European countries outside EU/EEA identified as groups with particularly low injury rates in both treatment levels. However, we also found notable differences in injury incidence between primary and secondary care in the immigrant population. Compared with non-immigrants, the relative risk of injury in immigrants was generally lower for injuries treated in primary care than in secondary care. In fact, immigrants from some regions (e.g., EU/EEA countries) and from some countries (e.g., Poland, Somalia) did not have a reduced risk of injuries treated in secondary care compared with non-immigrants, in contrast to their clearly lower risk of injuries treated in primary care. Moreover, this risk pattern varied by type of injury, as incidence rate ratios for immigrants were lower in primary care than secondary care for treatment of some injury types (superficial injuries, open wounds, burns) but not for others (fractures, dislocations/sprains/strains, poisoning).

Consistent with past research (Schwebel et al. 2005; Xiang et al. 2007; Laursen and Moller 2009; Sandvik et al. 2012; Norredam et al. 2013; Karimi et al. 2015; Saunders et al. 2017; Chang and Miller 2018; Andersen and Lauritsen 2020; Aamodt et al. 2020), we found that most immigrant groups had lower injury incidence than the non-immigrant population. One possible explanation for this finding is the so-called “healthy migrant effect”, which postulates that the arduous process of migration selects for better health amongst migrants than the average of both the population they leave behind and the population they enter (McDonald and Kennedy 2004). However, with increasing duration of stay in the host country, this health advantage gradually diminishes as a consequence of changes in lifestyle and adverse socioeconomic conditions (World Health Organization 2018). As poor health is a risk factor for injuries (Hong et al. 2013), the healthy migrant effect could help explain lower health care utilisation among newly arrived immigrants, but is less relevant for our findings of low injury incidence among immigrant groups that have lived in Norway for a long time (e.g., immigrants from Vietnam and Pakistan). Other potential explanations may include differences in risk-taking behaviour between the immigrant population and the host population, or different thresholds for seeking medical attention in the case of symptoms of illness and injury.

On its own, our register study cannot adjudicate between these alternative explanations. However, our finding that the difference in injury incidence between immigrants and non-immigrants was larger for treatment in primary care suggests that the threshold for seeking medical attention for injuries may be higher among immigrants. These injuries are usually less severe than injuries requiring specialist care, and are more susceptible to individual considerations about whether or not to seek medical treatment. However, this observed difference could also reflect language barriers or practical barriers in access to primary care, a general dissatisfaction with primary health care services, or poor knowledge and insufficient information about the organisation of the health care system. In support of the latter explanation, a previous Norwegian study reported lower utilisation of GPs but higher utilisation of EPHC among immigrants compared to non-immigrants (Diaz et al. 2015a), suggesting that some immigrant groups may use emergency services for non-urgent purposes (Norredam et al. 2004; Norredam et al. 2007; Credé et al. 2018). Most likely, differences in health care utilisation such as those suggested in our study are multifaceted and the product of a combination of factors.

Our findings also provide further documentation of the heterogeneity of the immigrant population, as injury incidence differed considerably between regions and countries of origin. In accordance with other studies (Aamodt et al. 2020; Saunders et al. 2018), we found particularly low incidence rates for immigrants originating from Asia (especially Southeast Asia), whereas rates were highest for immigrants from Western countries, which are culturally more similar to Norway. Our study also corroborates earlier findings from Scandinavian and other Western countries showing lower risk of poisoning (Saunders et al. 2017) and fractures (Aamodt et al. 2020) in immigrant groups, but higher risk of burns (Laursen and Moller 2009; Karimi et al. 2015; Elrod et al. 2019). Our analyses of the external circumstances in a smaller subset of injuries treated in secondary care reveal yet more heterogeneity in injury risk among immigrants. Consistent with past research, we found that immigrants had lower risk of injuries caused by self-harm than non-immigrants, but higher risk of injuries caused by assault (Norredam et al. 2013; Puzo et al. 2017; Tiruneh et al. 2019). Our study also adds to an extensive literature showing higher rates of occupational injuries among immigrants (Salvatore et al. 2013; Biering et al. 2017). Risk of sports injuries, on the other hand, was lower for immigrants than non-immigrants.

Explanation of such differences will likely need to invoke a variety of factors, including biological, cultural and social factors. Less substance abuse among immigrants (Adebe et al. 2015; Kjøllesdal et al. 2019) may partly help explain lower rates of poisoning, whereas lower risk of fractures could be mediated by differences in body composition or life-style factors such as nutrition and physical activity (Aamodt et al. 2020). Higher rates of occupational injuries among immigrants may be explained by a higher risk of occupational hazards, combined with language barriers and inadequate safety training (Moyce and Schenker 2018). Regardless of mechanisms, a novel feature of the present study is that many of these findings applied to treatment in both primary and secondary care, suggesting that differences according to immigrant status are robust and common for a wide range of injury severity.

Additional strengths of this study include the use of population-based registers and the ability to control for important sociodemographic confounders at the individual level. An important limitation, however, is that only immigrants eligible for residency (i.e., legal immigrants intending to stay in Norway for at least 6 months) were included in the study population. Consequently, this study does not include temporary or undocumented immigrants, who may be characterised by a different risk profile for injuries. For this reason, our findings do not necessarily generalise to the entire population of immigrants in Norway. Likewise, our analyses of the external circumstances in a smaller subset of injuries treated in secondary care are primarily based on data from the capital Oslo and may not be representative for the whole country.

Another limitation concerns our method of calculating incidence rates, which may not necessarily reflect the true incidence of injury. Specifically, we estimated the rates of individuals’ first health care contact with an injury diagnosis in the observation period separately for treatment in primary and secondary care. As some patients undergo treatment for the same injury in both primary and secondary care (i.e., as referrals or for follow-up treatment), some injuries were likely included in the numerator in incidence rates in both primary and secondary care, leading to an apparent overestimation of injuries. On the other hand, this method will not capture separate injury events occurring in an individual within the same treatment level during the observation period. For this reason, it is more precise to describe these estimates as rates of contact, as they reflect health care utilisation rather than true injury incidence. However, we do not expect that these sources of misclassification differ systematically according to immigrant status.

Potential extensions to the work reported in the current study include explorations of how immigrants’ use of primary and secondary care for treatment of injuries varies according to other factors known to affect health care utilisation, such as duration of stay in the host country and reason for migration (Saunders et al. 2018; McDonald and Kennedy 2004; Diaz et al. 2015a; Diaz and Kumar 2014; Norredam et al. 2014; Diaz et al. 2015b; Elstad 2016). Another avenue for future research involves assessing whether health care utilisation among immigrants differs by type of service (i.e., GPs vs. EPHC in primary care and inpatient vs. outpatient treatment in secondary care). In order to understand the mechanisms or pathways for the observed patterns in injury incidence, it would also be worthwhile to examine how differences in health care utilisation according to immigrant background is mediated by age, gender and other important sociodemographic variables. Finally, we do not know if our findings extend to children and older adults, nor how health care utilisation for treatment of injuries differs between immigrants and their descendants.

## Conclusions

In both primary and secondary care, health care utilisation for treatment of injuries in Norway was lower for immigrants compared to non-immigrants. Incidence rates were especially low for immigrants originating from Asia, Africa and European countries outside EU/EEA. Injury incidence among immigrants was lowest for treatment of fractures, poisoning, self-harm and sports injuries.

## Data Availability

The current study is part of a research project at the Norwegian Institute of Public Health. The datasets generated and analysed during this study are not publicly available due to data protection stipulations, but can be made available on a remote access platform to researchers who become project members. Alternatively, these data can be reconstructed anew by applying to each registry.

## Abbreviations

GP: General practitioner
EPHC: Emergency primary health care
EU: European Union
EEA: European Economic Area
ICPC-2: International Classification of Primary Care (2nd edition)
NPR: Norwegian Patient Registry
ICD-10: International Statistical Classification of Diseases and Related Health Problems (10th edition)
SES: Socioeconomic status
IRR: Incidence rate ratio
CI: Confidence interval
